# Spatial Transmission of 2009 Pandemic Influenza in the US

**DOI:** 10.1371/journal.pcbi.1003635

**Published:** 2014-06-12

**Authors:** Julia R. Gog, Sébastien Ballesteros, Cécile Viboud, Lone Simonsen, Ottar N. Bjornstad, Jeffrey Shaman, Dennis L. Chao, Farid Khan, Bryan T. Grenfell

**Affiliations:** 1 Department of Applied Mathematics and Theoretical Physics, University of Cambridge, Cambridge, United Kingdom; 2 Fogarty International Center, National Institutes of Health, Bethesda, Maryland, United States of America; 3 Department of Ecology and Evolutionary Biology, Princeton University, Princeton, New Jersey, United States of America; 4 Department of Global Health, George Washington University, Washington, D.C., United States of America; 5 Department of Entomology, Pennsylvania State University, State College, Pennsylvania, United States of America; 6 Department of Environmental Health Sciences, Mailman School of Public Health, Columbia University, New York, New York, United States of America; 7 Center for Statistics and Quantitative Infectious Diseases, Vaccine and Infectious Disease Division, Fred Hutchinson Cancer Research Center, Seattle, Washington, United States of America; 8 IMS Health, Plymouth Meeting, Pennsylvania, United States of America; Imperial College London, United Kingdom

## Abstract

The 2009 H1N1 influenza pandemic provides a unique opportunity for detailed examination of the spatial dynamics of an emerging pathogen. In the US, the pandemic was characterized by substantial geographical heterogeneity: the 2009 spring wave was limited mainly to northeastern cities while the larger fall wave affected the whole country. Here we use finely resolved spatial and temporal influenza disease data based on electronic medical claims to explore the spread of the fall pandemic wave across 271 US cities and associated suburban areas. We document a clear spatial pattern in the timing of onset of the fall wave, starting in southeastern cities and spreading outwards over a period of three months. We use mechanistic models to tease apart the external factors associated with the timing of the fall wave arrival: differential seeding events linked to demographic factors, school opening dates, absolute humidity, prior immunity from the spring wave, spatial diffusion, and their interactions. Although the onset of the fall wave was correlated with school openings as previously reported, models including spatial spread alone resulted in better fit. The best model had a combination of the two. Absolute humidity or prior exposure during the spring wave did not improve the fit and population size only played a weak role. In conclusion, the protracted spread of pandemic influenza in fall 2009 in the US was dominated by short-distance spatial spread partially catalysed by school openings rather than long-distance transmission events. This is in contrast to the rapid hierarchical transmission patterns previously described for seasonal influenza. The findings underline the critical role that school-age children play in facilitating the geographic spread of pandemic influenza and highlight the need for further information on the movement and mixing patterns of this age group.

## Author Summary

The determinants of influenza spatial spread are not fully understood, in part due to the insufficient geographic resolution of incidence data. We address this using a fine-grained private sector electronic health database of insurance claims data from health encounters in the US during 2009. We used physician diagnoses codes to generate a dataset of the weekly number of office visits with diagnosed influenza-like illness for 271 US locations. Applying statistical and mathematical models to these disease data, we find that the main autumn wave of the 2009 pandemic in the US was remarkably spatially structured. Its onset in the South Eastern US precipitated a slow radial spread that took 3 months to diffuse across the country. These patterns were replicated by models that included short-distance spatial transmission between nearby locations and increased transmission rates when school was in session. Our results contrast with previous modelling studies that indicated that environmental factors, population sizes, and long-distance transmission events (air traffic) are major determinants in disease spread. We conclude that the 2009 pandemic autumn wave spread slowly because transmissibility of the influenza virus was relatively low and children (who travel long distance far less than adults) were the predominant sources of infection.

## Introduction

Understanding the spatio-temporal spread of infectious disease is important both for design of control strategies and to deepen fundamental knowledge about the interaction between epidemic dynamics and spatial mixing of the host population. Dynamic models and statistical analyses have provided key insights into the spread of a number of acute, directly transmitted infections of humans, including measles, rotavirus, dengue, pertussis, and seasonal and pandemic influenza [1], [2], [3], [4], [5], [6], [7], [8], [9], [10]. A unifying feature of these analyses is the interaction of coupling between populations (often expressed in terms of ‘gravity’ or ‘radiation’ models for hierarchical spatial spread, [1], [2], [3], [5], [11], [12]) and demographic or environmental factors modulating transmission, in particular the seasonal aggregation of children in schools [13], [14], [15], [16], [17], or seasonal variation in humidity [18], [19]

Previous efforts have sought to forecast the likely spatial spread of pandemic influenza with model simulations accounting for intricate host demography and mixing data [10], [20]. However, a lack of finely resolved epidemiological data complicates validation and testing of such models. Analysis of long-term influenza-related mortality time series has highlighted the role of daily work commute as a driver of the regional spread of seasonal influenza in the US [3]. While mortality records were useful to explore the spatial transmission of the devastating 1918 pandemic in the US and UK [5], such data typically lack power to investigate disease patterns in small geographical areas or during more recent and milder seasons. However, increased disease surveillance and data availability in the context of the 2009 A/H1N1pdm09 pandemic provides a unique opportunity to explore the spatial spread of influenza in more detail, identify further data gaps, and validate existing models and theory. Here we used a rich dataset of influenza-like-illness records compiled from electronic medical claims and covering about 50% of outpatient physician visits in 2009 across the US to study influenza spread with an unprecedented level of detail. These electronic claims data have only recently been used for public health purposes, in particular to investigate the reduction in diarrhoea outpatient visits associated with Rotavirus vaccine introduction [21].

The 2009 pandemic spread rapidly across the world, soon after the putative emergence of the pandemic virus in Mexico [22]. The earliest laboratory-confirmed cases of pandemic influenza infection were reported in April 2009 in the South Western US. Subsequently, some cities, such as New York, Boston and Milwaukee, experienced intense community transmission in spring and summer [23], [24]. For most of the country however, there was no widespread outbreak until the autumn of 2009 when most pandemic-related deaths occurred [23]. Recent work has suggested that school fall terms starts were associated with the onset of the fall pandemic onsets in different US states [15], while reactive school closure in the spring reduced influenza transmission in Hong-Kong and Mexico [25], [26]. Another candidate driver of pandemic spread is low absolute humidity, which according to experimental and epidemiological studies may favour the transmission of influenza [18], [19], [27].

**Figure 1:**
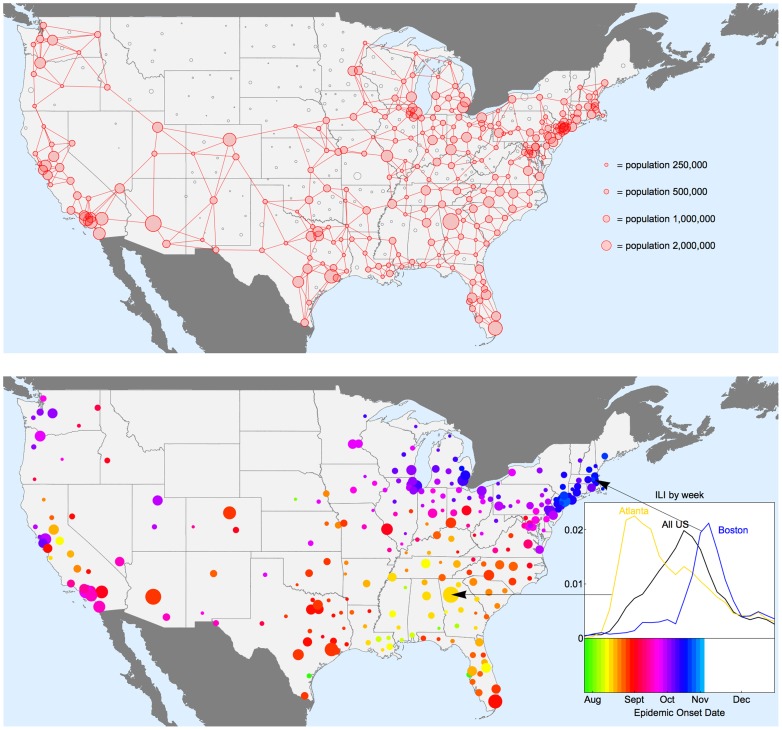
Geographic patterns of pandemic onset timings in studied locations in the 48 contiguous US states, autumn 2009. Upper panel: The map shows how the available influenza-like-illness (ILI) data are spatially stratified by 449 locations according to postal sectional center facility (SCF). The areas of the circles are proportional to population size. Locations in red are included in the analysis below, while those in black are excluded either due to small population size, or low reporting of ILI cases during 2009. See methods for neighbour network construction. Lower panel: Map of estimated timing of fall pandemic onset for the 271 locations with sufficient sampling for use in subsequent statistical and modelling analyses. These locations span 90% of the US population. There is a clear spatial spread visible for much of the US, with influenza pandemic onset earliest for the South Eastern states, and latest in the North East. Some places do not fit this overall pattern, and the distribution of timings on the west coast is more complex. *The inset plot* shows the proportion ILI during the fall wave of 2009 for the whole of the US aggregated (black), Atlanta (Yellow) and Boston (Blue): the aggregated ILI curve masks the relative sharp upswing in cases for individual locations as the pandemic onset timing differs considerably between locations.

To determine the relative contributions of population movements, demographics, school openings, prior immunity, and environmental factors to pandemic spread, we fit a series of mechanistic models to our highly resolved US influenza surveillance datasets [28]. To track pandemic activity, we compile weekly epidemic indicators of the number of influenza-like illness (ILI) patients stratified by zip code, providing disease information in 271 administrative areas, covering more than 90% of the US population in the 48 contiguous states (Figure 1, top panel). We focus on the dynamics of the fall wave of the 2009 H1N1pdm09 pandemic, as all sites experienced a clearly defined pandemic onset between July and November 2009.

## Results

Our analysis begins with a simple descriptive analysis of observed spatial autumn 2009 pandemic patterns and correlations with putative drivers. Armed with these empirical results, we construct a series of mechanistic epidemiological models to determine the importance of different processes for pandemic spread.

### Descriptive analysis

Our spatial analysis relies on the estimation of pandemic onset dates, which are based on the date when ILI incidence exceeded a seasonal threshold during summer-autumn 2009 [29], [30] (as most onset dates occurred in autumn, we refer to this pandemic wave as the “autumn wave” for the sake of simplicity; see methods for details). We disregard receipt of pandemic influenza vaccine as nearly all doses were administered after the onset of the autumn wave [31].

**Figure 2:**
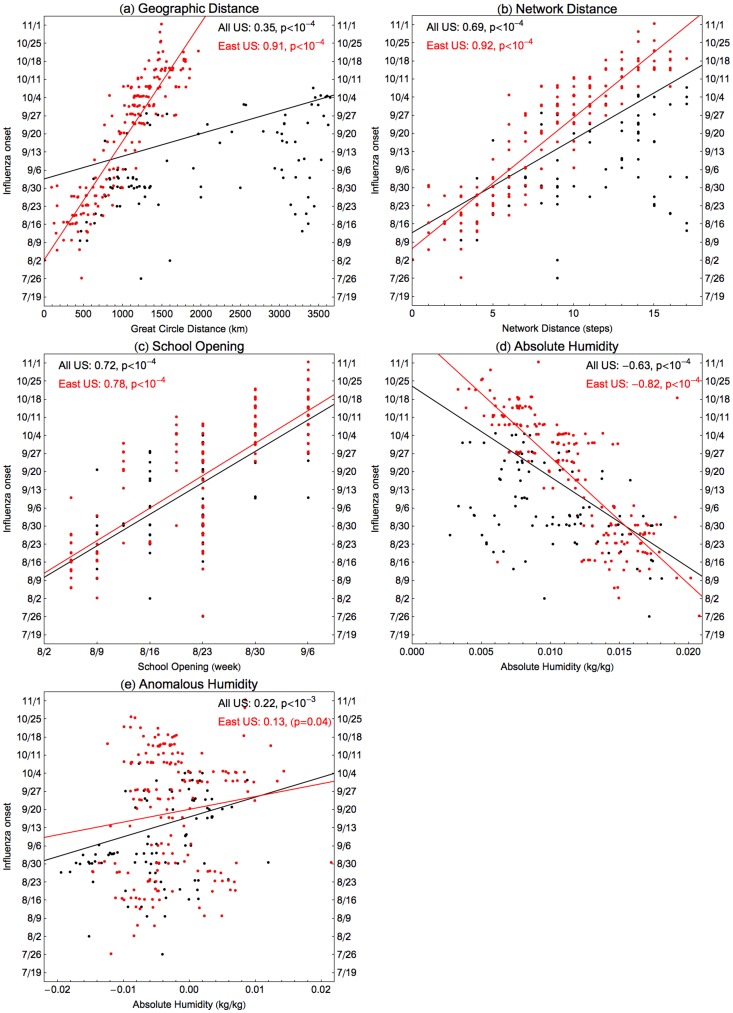
Univariate correlations between autumn 2009 pandemic onset timings and potentially contributing factors. The influenza onset timings are on the vertical axes for all four plots, and red points are locations in HHS regions 1–5 (East) and black in regions 6–10 (West). These are correlated either as East only (in red) or all US (in black) against four different candidate explanatory variables: (a) Distance from the earliest location in Alabama as measured by great circle geographic distance, (b) distance measured as minimum number of steps on the neighbour network, (c) the timing of fall school openings for the state and (d) absolute humidity and (e) humidity anomalies in the 7–10 days prior pandemic onset. See methods for details. Correlation coefficients and significance are inset in each plot. All of these correlations are highly significant (p<10^−4^).

Onset dates range between 26^th^ July to 1^st^ November, 2009 in the 271 locations, with a clear spatial patterning starting in South East US and spreading in all directions within around three months (Figure 1 and Supplementary Movie). Visually, the hub of the South Eastern spread is in Alabama or Georgia, and Dothan, Alabama had the earliest onset in these states (see also Figure S1). We correlate estimates of onset dates with four different putative drivers of spatial transmission: date of school term start [15], great circle distance from Dothan, distance on the nearest neighbour network from Dothan (see Figure 1b), and absolute humidity indicators (considering both raw values and anomalies in days 7–10 prior to pandemic onset, as in past work [18], [19]). Autumn pandemic onset is highly correlated with distance metrics and school starts (correlation coefficients 0.35–0.72, P<0.0001; Figure 2a–c) and moderately correlated with absolute humidity (coefficient −0.63, P<0.0001 for raw AH, and 0.22, P = 0.001 for anomalies; Figure 2d–e). Outliers in this correlation analysis may indicate a second important seeding event in California; hence correlations with distance are even stronger if restricted to the Eastern US (Figure 2 red points, correlation coefficient 0.91 and 0.92, P<0.0001). As AH in each location is generally decreasing through the autumn, the correlation between onset and AH at onset must be treated with some caution. However there is more signal here than can be explained just by the general temporal trend in AH: the correlation coefficient (−0.63) is stronger than that obtained from 10,000 random permutations of onset dates between locations.

Partial correlations were computed for each combination of predictors (Table 1). For the residuals from regression with geographic or network distance, weak but significant correlations were found with absolute humidity (coefficient −0.26 and −0.27, p<10^−4^) and schools (0.16, p = 0.02). For the residuals from school openings and both humidity measures, any of the other variables gave a typically moderate to high correlation (range of coefficients, 0.16–0.90, P<0.02). This finding suggests that a purely spatial process may dominate in explaining the timing of the autumn wave, perhaps modulated by environmental and school-related factors. However analysis of a more mechanistic epidemiological model is required to distinguish the relative contributions and interactions of these and other potential drivers.

**Table 1:** Partial correlations of putative factors affecting the onset of influenza autumn 2009 pandemic wave in 176 Eastern US locations.

	Geographic Distance	Network Distance	School Opening	Absolute Humidity	Anomalous Humidity
Geographic Distance	-	0.04 (p = 0.30)	**0.48 p<10^−4^**	**0.47 p<10^−4^**	**0.90 p<10^−4^**
Network Distance	0.09 (p = 0.12)	-	**0.49 p<10^−4^**	**0.49 p<10^−4^**	**0.91 p<10^−4^**
School Opening	**0.16 (p = 0.02)**	**0.15 (p = 0.03)**	**-**	**0.51 p<10^−4^**	**0.78 p<10^−4^**
Absolute Humidity	**−0.27 p<10^−4^**	**−0.26 p<10^−3^**	**−0.57 p<10^−4^**	**-**	**−0.85 p<10^−4^**
Anomalous Humidity	0.04 (p = 0.30)	0.08 (p = 0.15)	**0.16 (p = 0.02)**	**0.48 p<10^−4^**	**-**

Each of the five variables in the first row (geographic distance, network distance, school opening time, absolute humidity, humidity anomalies), residuals are computed from linear regression with the onset of influenza timings for locations in the East of the US. This table gives the correlation between these residuals and a second variable, listed in the first column.

For the residuals from regression with geographic or network distance (first two columns), weak correlation is found with absolute humidity (p<10^−4^) and schools (p = 0.02). For the residuals from school openings and both humidity measures (last three columns), any of the other variables give a significant correlation (p = 0.02 for one combination and p<10^−4^ for the other 11).

### Mechanistic epidemiological models

We build a simple spatial model for the spread of influenza, inspired by previous work on the 1918 pandemic [5] (see methods for full details). Briefly, treating each of 271 locations in the US as the statistical units, a maximum likelihood approach is used to fit the observed pandemic onset dates. The parametric model of the force of infection, the rate of outbreak initiation for each location, includes the contribution of both local and long-distance transmission. The outbreak in each location can be sparked by transmission from another nearby location: this contribution to the force of infection is modelled using a power law kernel driven by population size and distance (hereafter referred to as the gravity model) [1], [3], [5], [12]. Alternative spatial kernels based on different model formulations or distance metrics were also explored, including Gaussian kernel and grid distance (methods). Further, we introduced a normalization parameter that quantifies how connectivity may depend on the number and size of neighbouring populations, following [5], akin to the difference between density-dependent and density-independent transmission [32]. In addition to short-range disease transmission, a term was included to account for the background probability of an outbreak spark (hereafter referred to as external seeding), which could be seeded by imported infections from distant locations (domestically or internationally) or even a low-level persistent local chain of infection that survived the summer. Both external seeding and local transmission were also allowed to depend on whether or not schools were in session and also to scale according to population sizes to some power. The force of infection was also allowed to be modulated by previous immunity to pandemic A/H1N1pdm09 (as measured by indicators of the intensity of the spring 2009 pandemic wave) and absolute humidity. We evaluate every possible combination of these factors to explain pandemic onset dates using the corrected Akaike information criteria.

**Figure 3:**
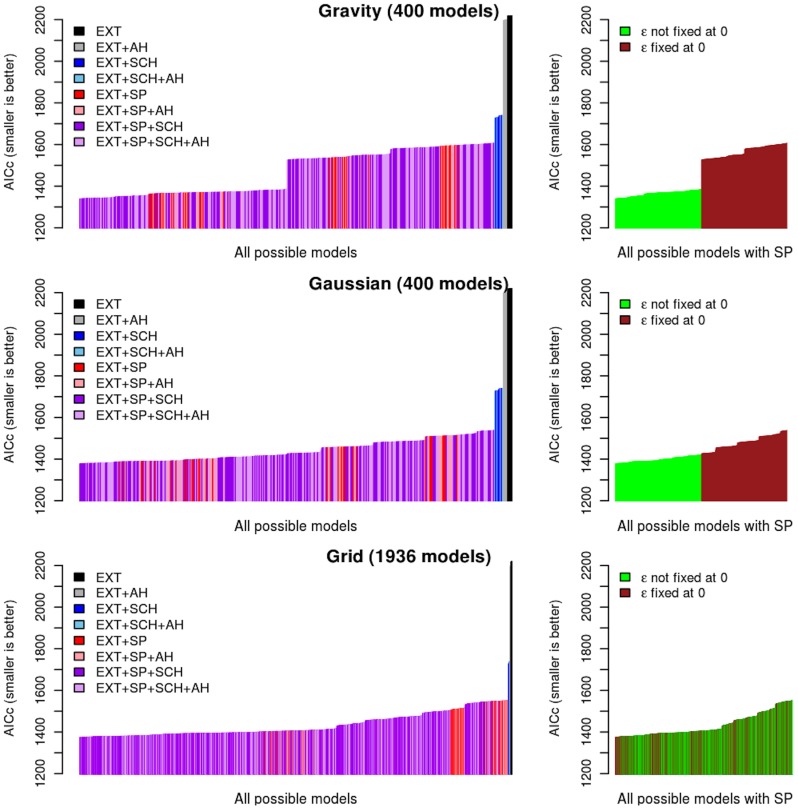
Parsimony of model fits to the autumn 2009 pandemic onset timings – corrected Akaike information criteria (AICc) histograms for all models. Left panels: AICc per categories (EXT: External seeding; AH: Absolute Humidity; SCH: Schools; SP: Space). Each vertical line represents one possible model. Right panels: AICc for models containing parameters related to space (SP) segregated regarding the assumption made on density dependence in connectivity between SCFs.

Figure 3 shows the relative importance of each factor and their interactions. Irrespective of the spatial kernel used, introducing terms for schools and/or spatial spread results in a significant improvement of fit over models exclusively considering external seeding. Models including spatial spread alone result in better fit than models purely driven by schools terms. For both the gravity and Gaussian models, the normalisation described above consistently improves the fit, whereas it is less important for the grid models. Of the spatial kernels tested, the gravity model offers the best fit (AICc more than 30 lower than other kernels, see Table S1). In line with a strong distance effect evidenced in the correlation analysis, the distance exponent of the best-fit gravity model was high (2.6, See Table S2) while the population size exponent was low (0.27).

Overall, the best model includes terms for spatial transmission, the effect of schools on spatial transmission, and a baseline external seeding rate (see Table 2). The external seeding rate was small but not zero (Table S2), and only appears to have played any significant role in a small number of locations (Figure S1). Neither absolute humidity nor prior exposure to influenza during the spring wave were part of the best model. Taken together, these results support a scenario where autumn pandemic onset is determined by a local mode of spatial spread, which is density-independent and enhanced by schools being in session.

**Table 2:** Parsimony of model fits to the autumn 2009 pandemic onset timing: best AICc for each model category.

Model Category				Log Likelihood	Number of parameters	AICc	ΔAICc
EXT				−1106.65	2	2217.35	879.39
EXT			+AH	−1094.77	3	2195.63	857.67
EXT		+SCH		−860.77	3	1727.64	389.68
EXT		+SCH	+AH	−859.12	4	1726.40	388.44
EXT	+SP			−675.06	5	1360.34	22.38
EXT	+SP		+AH	−675.04	6	1362.04	24.08
**EXT**	**+SP**	**+SCH**		**−662.82**	**6**	**1337.96**	**0**
EXT	+SP	+SCH	+AH	−662.16	7	1338.75	0.79

Each row corresponds to best fit of the most parsimonious model in a given category. The categories are the eight that include EXT (external seeding) plus all possible combinations of AH (absolute humidity), SCH (effect of schools) and SP (spatial transmission). The row in bold indicates the most parsimonious model, as determined by AICc, and ΔAICc gives the difference from the AICc of this model. For each of the categories including SP, the most parsimonious model used the gravity kernel. Table S1 gives an extended version of this table with the different spatial kernels tested separately.

One-step ahead predictions (Figure S2) and full simulations (Figure S3) confirmed that the best model could broadly reproduce the observed spatial dynamics of autumn pandemic wave onsets. However the model predicts later onsets than those observed in California's Central Valley, again suggesting multiple seeding events (Figure S1). Simulating the effect of setting schools to be permanently closed, the general spatial structure of the wave was similar to observed, but spread was substantially slower (Figure S3).

## Discussion

To our knowledge, this is the most detailed analysis to date of the local and regional dynamics of influenza pandemic spread, made possible by the availability of rich electronic disease datasets maintained in the private sector. Our analysis shows that the spread of the A/H1N1pdm09 pandemic during autumn 2009 in the US was highly spatially structured, with a clear wave originating in the South Eastern region of the country and slowly spreading outwards over a 3-month period. Variation in school openings alone cannot explain the observed fine grain variations in pandemic onset across the US, but school opening does exert a significant effect on the spread of the pandemic, consistent with past research [15], [25], [26], [33]

It is remarkable that the main 2009 pandemic wave, set in an era of intense air traffic and regional ground transportation, showed such a short-range mode of spread – so local that observed outbreak patterns conflicted with the usual model of rapid transmission between distant major cities followed by spread to less populated areas [3], [10], [20]. This intriguing picture of mainly local spread could be due to a combination of two factors: the relatively low transmissibility of the 2009 A/H1N1pdm09 virus [34] and the importance of children in pandemic spread [35]. In turn, both of these factors could be consequences of a strong build-up of anti H1N1pdm09 immunity in older cohorts due to earlier exposure to related viruses [25], [36]. The global transportation network likely played a significant role in the initial spread of the pandemic virus in spring 2009 [37], [38], [39].

Analysis of long-term mortality data indicates that the regional spread of seasonal influenza is driven by longer-range commuter-driven movements of adults with strong dependence on population sizes of recipient and donor locations [3]. Previous models of pandemic spread make similar assumptions [10], [20]; however, our analysis of detailed local morbidity data suggest that the travel patterns of *school-age* children may be a major factor explaining the spread of the 2009 autumn pandemic wave in the US. While intuitively one might expect that movements of children are typically shorter-range and revolve around home and school, limited information exists on contact rates in this age group. The 2009 experience underlines the urgency for improved understanding of the dynamics of epidemiologically-relevant spatial and social mixing in children.

The relatively modest transmissibility of the A/H1N1pdm09 virus, with an effective reproduction ratio estimated at around 1.5 [34], might also explain why long range travel was a lesser determinant of the spread of the pandemic. With a low reproduction ratio, occasional long-range imports of infection may die out after a small number of generations of transmission, and hence simply fail to “take”. In contrast, a large outbreak in a proximate community will result in repeated infection challenges, and inevitably a successful chain of infection will commence. Intriguingly, the effective reproduction number of seasonal influenza is typically lower than that of the A/H1N1pdm09 virus, and hence we would expect an even more localized and slower spread for seasonal outbreaks than for the autumn 2009 pandemic. Unfortunately, no epidemiological data at a comparable level of spatial detail is available for comparison. Further, as hypothesized in earlier work, the transmission patterns of seasonal influenza epidemics may not be predictive of pandemic patterns, due to differences in outbreak timing and age distribution of infection [35], [40], [41], [42]. Understanding the relative contribution of virus transmissibility, seasonality, and mean age at infection on the spatial dynamics of influenza is an interesting area for future work.

Another surprising feature of the 2009 autumn pandemic wave was the late arrival in large northeastern cities – regardless of whether these cities had experienced an early summer wave. For instance, the five boroughs of New York City suffered a major spring pandemic outbreak and were particularly late in experiencing an autumn outbreak, in contrast to the less densely populated cities upstate. This implies that chains of influenza transmission from the spring wave did not persist over the summer in most places, consistent with phylogeographic analysis of A/H1N1pdm09 viruses suggesting reintroduction of a single dominant viral lineage in the autumn in the US [24]. This phylogenetic pattern is not repeated in all countries, for example in Scotland [43], and synthesising the evolutionary and epidemiological observations for influenza spatial transmission is proving challenging [44]. It would therefore be interesting to test whether other countries also experienced slow and localized pandemic transmission and how that correlates with the corresponding observed evolutionary patterns.

Although our study is the first to investigate influenza spread at such a high level of spatial resolution and over such a broad geographic area, our findings may be compared with those of an earlier study of the 1918 pandemic in the US, England and Wales by Eggo *et al.* [5], made using a similar modelling framework. Interestingly, both studies suggest that transmission should be normalised by a weighted sum of all populations, meaning that transmission is nearly density-independent. However the fitted spatial kernels differ between the studies: in the study of the 1918 datasets [5] spatial transmission scales approximately as distance to the power −1, whereas in this 2009 study the distance exponent is around −2.6, implying much sharper localised transmission. Differences in spatial resolution of the data available and transmissibility of the 1918 and 2009 pandemic virus may explain these conflicting results. Clearly, more high resolution analyses of influenza disease spread are needed, in a variety of geographic settings, before the spatial transmission of pandemic influenza can be accurately predicted.

Several caveats are worth noting in our study. Here we have developed time series data of influenza-like illness coded by physicians (as a proxy of H1N1pdm09 activity), but these patients were not generally laboratory-confirmed. The contribution of other respiratory pathogens to influenza-like illness diagnoses is likely small in all age groups given the unusual timing of the pandemic outbreak during autumn when other important respiratory viruses – especially respiratory syncytial virus – are typically not epidemic. Further, because of its unusual timing, the onset of the pandemic was relatively easy to identify at a fine spatial and temporal resolution, given low background of respiratory diagnoses.

Our analysis was limited to the 2009 pandemic autumn wave period, and it would be interesting to model the spread of seasonal influenza epidemics at the scale studied here, although outbreak onset dates would be more difficult to identify. Additionally, we did not explore the spatial patterning of the 2009 spring wave, because its presence in the US was much more sporadic than the autumn wave and onset dates could be obscured by changes in health-seeking behaviour as people become aware of the new pandemic. Further, even though the 271 locations in our study represent 90% of the US population, we had to exclude cities with less than 200,000 inhabitants due to demographic noise. Most of these cities are located in the central US, a less well-connected and potentially interesting region that was not considered here. In addition, we used school term data at the state level [15], rather than at the county or city-level, given that detailed school data are not publicly available in most states. Further, our models do not integrate any age information, although analysis of age-stratified disease incidence time series revealed very similar patterns of pandemic onsets in the 271 US locations (not shown). Finally, we did not consider radiation models of spatial flux [11]: these are unlikely to add significantly to the present analysis as the picture of sequential outbreak onset is so clear already, and a normalisation factor has been included in the gravity formulation to account for the varying population density across the US. However it would be interesting to test the relative performance of radiation and gravity models on a finer grained influenza data set, particularly if a matching resolution of school data were available.

Overall, our results are robust: they do not depend on the exact formulation of the spatial model nor the definition of epidemiologic indicators. Our conclusions highlight the role of local transmission in the spread of the major autumn 2009 pandemic wave. This work highlights the importance of *testing* model predictions against detailed empirical disease data and suggests that fine-scale transmission models should take these results into account for simulation of future pandemic outbreaks. As ever, a synthesis of models, demographic, viral sequence data, environmental and movement data with *multiple* incidence data sets collected by different means would offer a particularly powerful way forward to understand infectious disease dynamics and improve preparedness for outbreaks of novel respiratory pathogens.

## Methods

### Data source

Weekly time series of outpatient visits for influenza-like-illness and total visits were compiled from the visit-level database of CMS-1500 medical claims data maintained by IMS Health, which captures a convenience sample of about half of all physician visits in the US. We first developed and employed a case definition of influenza-like illness (ILI) as any mention of a diagnostic code for influenza (ICD487x-488x) OR [fever and (sore throat or cough), (ICD780.6 and (462 or 786.2)] OR febrile viral illness (ICD079.99). Most of the cases were coded as ICD9 = 079.99 rather than the influenza specific code 487–488 – which probably reflects that only few doctor's offices utilized rapid testing for H1N1pdm09 influenza, on advice from the CDC and WHO to allow the laboratories to focus supplies and effort on severe cases only [45].

We extracted the weekly number of visits that met the ILI definition and also total number of all visits, stratified by 3-digit zip code of the physician's office. The IMS database covered 906 three-digit zip codes in the continental US during the 2009 pandemic period. The resulting syndromic case definition was validated against CDC's ILI surveillance system at the national and HHS regional level for seasonal and pandemic seasons; furthermore the ILI time series displayed known geographical heterogeneities, in particular large early summer waves in Northern cities like New York City but an absence of such patterns in upstate New York and the South [46].

### Standardization of ILI data

The three-digit zip codes were aggregated according to 449 “sectional center facility” (SCF) as defined by the United States Postal Service, to make geographically meaningful population divisions. The case definition was sensitive enough to yield a large number of weekly cases in most SCFs year-round; however both coverage and reporting rate may vary by location and time. To generate stable time series, we used the ratio of ILI to total number of visits. We restricted the analysis to the continental US, to SCFs with populations of more than 200,000, and to SCFs with more than 250 ILI cases reported in 2009. This reduced the total number of SCFs available for analysis to 271 but still accounted for over 90% of the US population. These SCF are shown in Figure 1 (top panel), and we refer to these as “locations” in the main text.

### Geographic data

Population numbers were determined from US Census 2000 data and zip codes weighted by population size to determine SCF centres. The eastern US was defined as HHS regions 1–5 [47]. The neighbour network (also called ‘grid model’) was constructed by joining each location to its four nearest neighbours and allowing all links to be reciprocal. The median school opening date was used for each state, and methods for collecting these data are given in Chao *et al.* 2010 [15]. The absolute humidity data were daily 2 m above-ground specific humidity conditions compiled from the North America Land Data Assimilation System (NLDAS) project, 1999–2009 [48]. For each SCF, we calculated the AH values and AH anomalies in days 7–10 prior pandemic onset, where daily AH anomalies are defined as observed AH minus average AH for the same day of the year during 1999–2008.

### Definition of influenza pandemic onset

Weeks of national “low ILI activity” between 2001–2009 for aggregated US are defined as when the ILI ratio is below 0.6%, and a sinusoid is fitted to these weeks to determine the phase (similar to methods used for mortality data [29], [30]). Using this phase and the same set of weeks, amplitude plus a quartic function of time is fitted to the ILI ratio for each SCF to give an approximate seasonal baseline. As the pandemic does not necessarily respect the usual annual timing of influenza, we define a conservative pandemic threshold as the maximum of the sinusoidal model baseline during 2009 plus a small additional buffer (0.2%). Using absolute numbers for 2009, a binomial test (with exact probabilities) is used to determine if the observed number of visits is significantly (p<0.01) above threshold in each week. If there are at least three consecutive weeks that are significantly above threshold, then the first such week is considered to be the week of pandemic onset. To interpolate to a slightly greater degree of resolution, we estimate the number of ILI visits and total visits in the half week before onset using the geometric mean, and the binomial test is again used to determine if the fall wave start time should be moved back by half a week. For the fall wave of 2009, the calculated pandemic onset timings will not be sensitive to these methods of calculation as the epidemic upswing in each location is so sharply defined.

### Transmission model

We use a maximum likelihood approach based on a simple mechanistic model. Following Eggo *et al.* [5], the probability that the fall wave starts at time 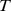 for a given location (indexed by 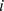) is given by
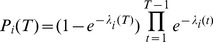
where 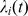 is the force of infection at location 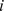 at time 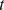. For the gravity model, this force of infection is given by
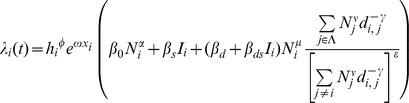
where the following are explanatory variables: 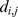 is the great circle distance between locations 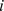 and 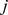, and for each location 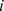: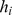 is the absolute humidity, 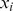 is the intensity of the spring wave (percentage of total pandemic excess ILI cases that were reported during the spring), 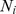 is the population size normalised by average population size, 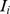 is an indicator function of time which is 1 when schools are open and 0 otherwise. 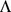 is the set of indices of currently infected locations.

The estimated parameters are as follows: 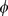 and 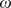 are the effects of the spring wave and humidity in modulating the full transmission rate. The 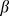 parameters are all transmission rate factors: 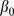 is for the background rate of infection (including external seeding from domestic and international locations and local chains of transmission surviving over summer), 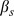 is a boost due to schools being in, 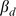 is the spatial transmission coefficient and 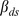 is the boost to spatial transmission due to schools being in. 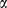, 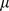 and 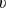 are all exponents on population size, representing the effect of population size on the background rate of infection, for recipient population and donor population in spatial transmission respectively. The distance exponent in the spatial kernel of the gravity model is 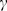. For the spatial transmission, the numerator of the fraction is the sum over infected locations (weighted by distance and population size to some powers), and the denominator is the same sum but over all locations. The denominator is to the power 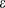: so 0 corresponds to no normalisation (fully density-dependent), and 1 corresponds to full normalisation (fully density-independent).

For the Gaussian model, the expression is the same except for the form of dependence on distance and the parameter 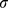 gives the distance scaling in the Gaussian (and 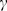 is no longer used):
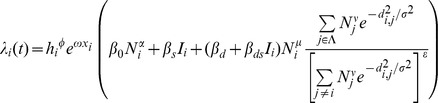



The grid model is slightly different as instead of explicit dependence on geographic distance, we use the set of locations one step (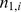) or two steps (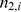) away for location 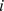, as defined by the constructed grid:
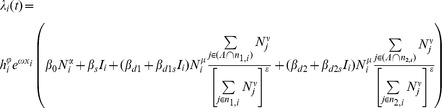



There is no distance parameter, but now the spatial transmission parameters have been extended from two to four to account for transmission to locations one and two steps away: 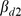, 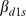, 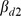 and 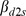.

Simpler models can be made from all these spatial kernels by “turning off’ combinations of parameters, i.e. setting them to zero. This makes 400 gravity, 400 Gaussian and 1936 grid models. For each of the 2736 models, likelihood was maximised using the Nelder-Mean simplex algorithm as implemented in the GNU scientific library in C. Simulations were done in C using the ranlux algorithm of Lüscher with maximum luxury level as provided by the GSL library. Convergence was assessed by likelihood profiles (Figure S4).

## Supporting Information

Figure S1 **Analysis of contribution of external seeding by location.** Upper panel: The percentage contribution of external seeding to the model force of infection was calculation for each location at the observed date of pandemic onset. For 253 out of 271 locations the external seeding accounted for less than 5% of the force of infection, indeed in 177 it accounted for less than 1% of the force of infection. On the map, locations with 5–10% external contribution are marked in yellow (five locations), 10–50% in orange (seven locations, and all less than 25%), and those over 50% in red. Lower panel: Plots correspond to individual locations: the black curve gives the standardized ILI (y-axis) against date (x-axis) for the calendar year 2009, the grey curve and shaded area give the pandemic onset threshold, and the blue line marks the calculated autumn pandemic onset date (see methods for full details). The plot labels give the location name an in brackets the percentage of force of infection at week of onset that is contributed by external seeding. The first row gives three representative plots (Atlanta GA, Boston MA and Trenton NJ). In each case, the contribution to force of infection at week of onset was less than 5%, and it can be seen that the week of onset can be identified unambiguously. The second row shows three of the six places where external contribution at week of onset was over 50%, but date of onset is not clearly identified. St. Petersburg FL, the standardized ILI dips below threshold again after onset is detected. For Corpus Christi TX there is also a dip after onset, though it does not drop below threshold. For Grand Island NE, there is also a dip after onset, and the general rise of ILI is not as sharp as other locations. If week of onset for a location is misidentified as earlier than other locations nearby, then the apparent dominant contribution of external seeding would be artefactual: we cannot exclude this for these three locations. The third row shows the remaining three places where external contributions was over 50%. In each of these, the week of onset is clearly defined. Visually from the supplementary movie, both Dothan AL and Stockton CA appear to be at or near the source of clear regional waves, so these are likely to correspond to true external seeding events. The final location, Baltimore MD, does not appear to be the origin of separate regional wave, and the apparent high contribution of external seeding can be explained as an artefact: there is another location very close by (Linthicum MD). The power-law dependence on distance in the transmission model and the normalisation mean that essentially these two locations only “see” each other. It happens that Baltimore onset is a week before Linthicum, so this appears as having a strong component of external contribution to the force of infection. This highlights a potential weakness of gravity normalisation methods, but could be overcome by modifications such as merging locations that are close together, or by capping the power-law dependence on distance within a certain range. In this model, the external seeding term ameliorates the difficulty. In summary: the evidence here suggests only two likely external seeding events: near Dothan AL and near Stockton CA.
(PDF)

Figure S2 **Comparison of most parsimonious models to observed onset timings of the autumn 2009 pandemic.** A: Conditional probability of epidemic onset, model prediction and residual analysis for the most parsimonious model per category. For each location (sorted by increasing time of onset) probabilities of epidemic onset conditional on the previous disease dynamic are represented in grey scale with mode in brown and model prediction (average) in orange. Residuals are computed as the difference between the model prediction (brown) and the data (red) and reported on the maps. Titles indicate parameters defining these models. Models discarding local spatial transmission are unable to reproduce the qualitative patterns of spread (upper panels). The inclusion of local spatial spread with or without school opening means the model broadly tracks the spatial progression of pandemic onsets (lower panels). The best-fit model is able to reproduce the general spread pattern of the autumn 2009 pandemic wave originating from the South Eastern US, but the residuals are geographically clustered, particularly in California and Florida. Most notably, the model predicts later onsets than those observed in California's Central Valley and earlier onsets than observed in Florida. B: While the spatial model with and without schools give broadly similar visual results, there is a significant improvement in model fit (see Table 2), and here this difference is investigated by location. For each location (x-axis and map) difference of log likelihood (conditional on the previous disease dynamic) between the model with and without schools are reported.
(PDF)

Figure S3 **Predictions from the most parsimonious model on the effect of school closure on pandemic onset timings of the autumn 2009 pandemic in the US.** 1000 realisations were started without any locations infected. The full model (black lines) was simulated using maximum likelihood parameters of the most parsimonious model (see table 2). The simulations without school opening (grey lines) uses the same parameters, but schools were set as closed. The general spatial structure of the wave is similar to observed or simulated with the correct school opening times, but the spread was substantially slower. However, the exact length of the delay was sensitive to the fitted parameters. In the lower graphs, the spatial transmission parameter (β_d_) was fixed, other parameters refitted and the above simulations repeated. The 95% confidence intervals are indicated by black dashed lines and the maximum likelihood estimate by orange lines. The lower left plot shows the distribution of the time when 50% of the locations were infected (T50). The simulated times with schools (black boxes) was not sensitive to the fixed parameter, but the extra delay with schools closed (grey boxes) was highly variable over the confidence interval. The lower right graph shows how the transmission parameters are interdependent, which explains the sensitivity in simulation. In summary: the extent of the epidemic slowing from closing schools is difficult to estimate accurately from this model, but is likely to be substantial.
(PDF)

Figure S4 **The profile likelihoods for the parameters in the most parsimonious model.** For the transmission rate parameters (top row), logged parameter values are used, while the exponents (bottom row) are given unlogged. The orange line marks the maximum likelihood, and the dotted lines give the range for a drop of 1.92 in the log likelihood, corresponding to a 95% confidence interval.
(PDF)

Table S1 **Most parsimonious model per category including different spatial kernels.** An extension of Table 2 from the main text: this table gives the log likelihood and AICc for the maximum likelihood fits to the most parsimonious models in each category. Here, additional results are given for the alternative spatial kernels (Gaussian, or using grid distance). For each model category, the most parsimonious model is specified by the parameters that are non-zero, which are given in the final column. In all cases, the gravity model has much lower AICc than the Gaussian or the grid models. Despite the crudeness of the spatial grid, the grid model performs surprisingly well.
(PDF)

Table S2
**Fitted parameters for the most parsimonious model.** The most parsimonious model has six non-zero parameters. These are given in the table together with their maximum likelihood values and confidence intervals, as determined by a drop of 1.96 in the profile likelihoods. Setting the other parameters to zero, the force of infection for location *i* can be written as: 
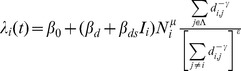
. This force of infection is a rate and the units correspond to the time step Δ*t* = half week.(PDF)

Movie S1 **Influenza-like-illness in the US from January 2009 to April 2010.** The area of the disc on each location is proportional to the population size and the colour represents the standardised ILI (see methods for details). The lower panel shows standardised ILI for the aggregate of all locations.
(MOV)
