# Supplementary material for: Spatial Transmission of 2009 Pandemic Influenza in the US
Source: PLoS Comput Biol. 2014 Jun 12;10(6):e1003635. doi: 10.1371/journal.pcbi.1003635 (PMC4055284; doi:10.1371/journal.pcbi.1003635)
Supplement: Figure S1 — Analysis of contribution of external seeding by location. Upper panel: The percentage contribution of external seeding to the model force of infection was calculation for each location at the observed date of pandemic onset. For 253 out of 271 locations the external seeding accounted for less than 5% of the force of infection, indeed in 177 it accounted for less than 1% of the force of infection. On the map, locations with 5–10% external contribution are marked in yellow (five locations), 10–50% in orange (seven locations, and all less than 25%), and those over 50% in red. Lower panel: Plots correspond to individual locations: the black curve gives the standardized ILI (y-axis) against date (x-axis) for the calendar year 2009, the grey curve and shaded area give the pandemic onset threshold, and the blue line marks the calculated autumn pandemic onset date (see methods for full details). The plot labels give the location name an in brackets the percentage of force of infection at week of onset that is contributed by external seeding. The first row gives three representative plots (Atlanta GA, Boston MA and Trenton NJ). In each case, the contribution to force of infection at week of onset was less than 5%, and it can be seen that the week of onset can be identified unambiguously. The second row shows three of the six places where external contribution at week of onset was over 50%, but date of onset is not clearly identified. St. Petersburg FL, the standardized ILI dips below threshold again after onset is detected. For Corpus Christi TX there is also a dip after onset, though it does not drop below threshold. For Grand Island NE, there is also a dip after onset, and the general rise of ILI is not as sharp as other locations. If week of onset for a location is misidentified as earlier than other locations nearby, then the apparent dominant contribution of external seeding would be artefactual: we cannot exclude this for these three locations. The third row shows the [file pcbi.1003635.s001.pdf]

Figure S1

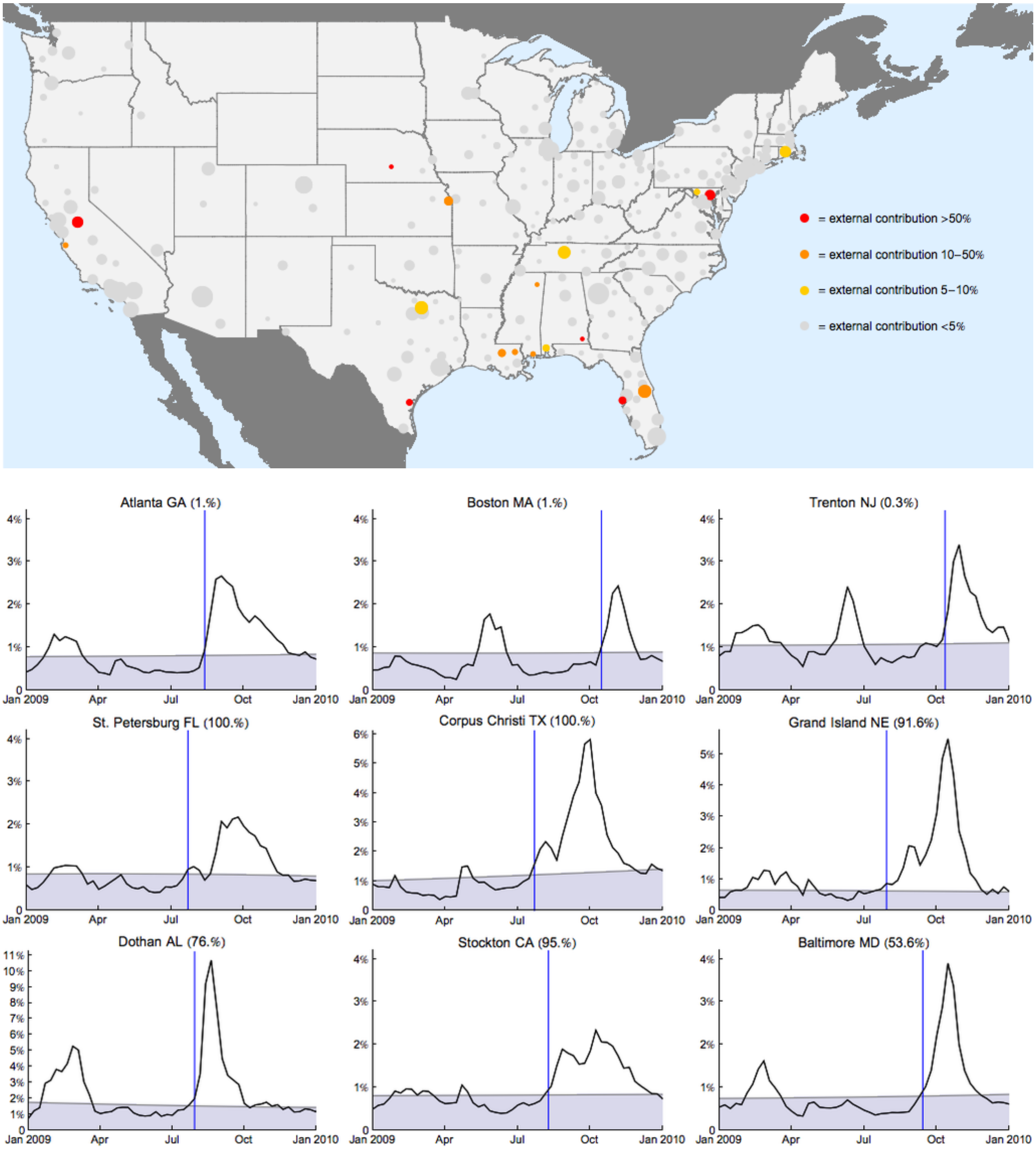
= external contribution >50%
= external contribution 10−50%
= external contribution 5−10%
= external contribution <5%
Atlanta GA (1.%)
Boston MA (1.%)
Trenton NJ (0.3%)
St. Petersburg FL (100.%)
Corpus Christi TX (100.%)
Grand Island NE (91.6%)
Dothan AL (76.%)
Stockton CA (95.%)
Baltimore MD (53.6%)
Jan 2009
Apr
Jul
Oct
Jan 2010

Analysis of contribution of external seeding by location

Upper panel:
The percentage contribution of external seeding to the model force of infection was calculation for each location at the observed date of pandemic onset. For 253 out of 271 locations the external seeding accounted for less than 5% of the force of infection, indeed in 177 it accounted for less than 1% of the force of infection. On the map, locations with 5-10% external contribution are marked in yellow (five locations), 10-50% in orange (seven locations, and all less than 25%), and those over 50% in red.

Lower panel:
Plots correspond to individual locations: the black curve gives the standardized ILI (y-axis) against date (x-axis) for the calendar year 2009, the grey curve and shaded area give the pandemic onset threshold, and the blue line marks the calculated autumn pandemic onset date (see methods for full details). The plot labels give the location name an in brackets the percentage of force of infection at week of onset that is contributed by external seeding.

The first row gives three representative plots (Atlanta GA, Boston MA and Trenton NJ). In each case, the contribution to force of infection at week of onset was less than 5%, and it can be seen that the week of onset can be identified unambiguously.

The second row shows three of the six places where external contribution at week of onset was over 50%, but date of onset is not clearly identified. St. Petersburg FL, the standardized ILI dips below threshold again after onset is detected. For Corpus Christi TX there is also a dip after onset, though it does not drop below threshold. For Grand Island NE, there is also a dip after onset, and the general rise of ILI is not as sharp as other locations. If week of onset for a location is misidentified as earlier than other locations nearby, then the apparent dominant contribution of external seeding would be artefactual: we cannot exclude this for these three locations.

The third row shows the remaining three places where external contributions was over 50%. In each of these, the week of onset is clearly defined. Visually from the supplementary movie, both Dothan AL and Stockton CA appear to be at or near the source of clear regional waves, so these are likely to correspond to true external seeding events.

The final location, Baltimore MD, does not appear to be the origin of separate regional wave, and the apparent high contribution of external seeding can be explained as an artefact: there is another location very close by (Linthicum MD). The power-law dependence on distance in the transmission model and the normalisation mean that essentially these two locations only “see” each other. It happens that Baltimore onset is a week before Linthicum, so this appears as having a strong component of external contribution to the force of infection. This highlights a potential weakness of gravity normalisation methods, but could be overcome by modifications such as merging locations that are close together, or by capping the power-law dependence on distance within a certain range. In this model, the external seeding term ameliorates the difficulty.

In summary: the evidence here suggests only two likely external seeding events: near Dothan AL and near Stockton CA.
