# Supplementary material for: Spatial Transmission of 2009 Pandemic Influenza in the US
Source: PLoS Comput Biol. 2014 Jun 12;10(6):e1003635. doi: 10.1371/journal.pcbi.1003635 (PMC4055284; doi:10.1371/journal.pcbi.1003635)
Supplement: Figure S2 — Comparison of most parsimonious models to observed onset timings of the autumn 2009 pandemic. A: Conditional probability of epidemic onset, model prediction and residual analysis for the most parsimonious model per category. For each location (sorted by increasing time of onset) probabilities of epidemic onset conditional on the previous disease dynamic are represented in grey scale with mode in brown and model prediction (average) in orange. Residuals are computed as the difference between the model prediction (brown) and the data (red) and reported on the maps. Titles indicate parameters defining these models. Models discarding local spatial transmission are unable to reproduce the qualitative patterns of spread (upper panels). The inclusion of local spatial spread with or without school opening means the model broadly tracks the spatial progression of pandemic onsets (lower panels). The best-fit model is able to reproduce the general spread pattern of the autumn 2009 pandemic wave originating from the South Eastern US, but the residuals are geographically clustered, particularly in California and Florida. Most notably, the model predicts later onsets than those observed in California's Central Valley and earlier onsets than observed in Florida. B: While the spatial model with and without schools give broadly similar visual results, there is a significant improvement in model fit (see Table 2), and here this difference is investigated by location. For each location (x-axis and map) difference of log likelihood (conditional on the previous disease dynamic) between the model with and without schools are reported. (PDF) [file pcbi.1003635.s002.pdf]

Figure S2

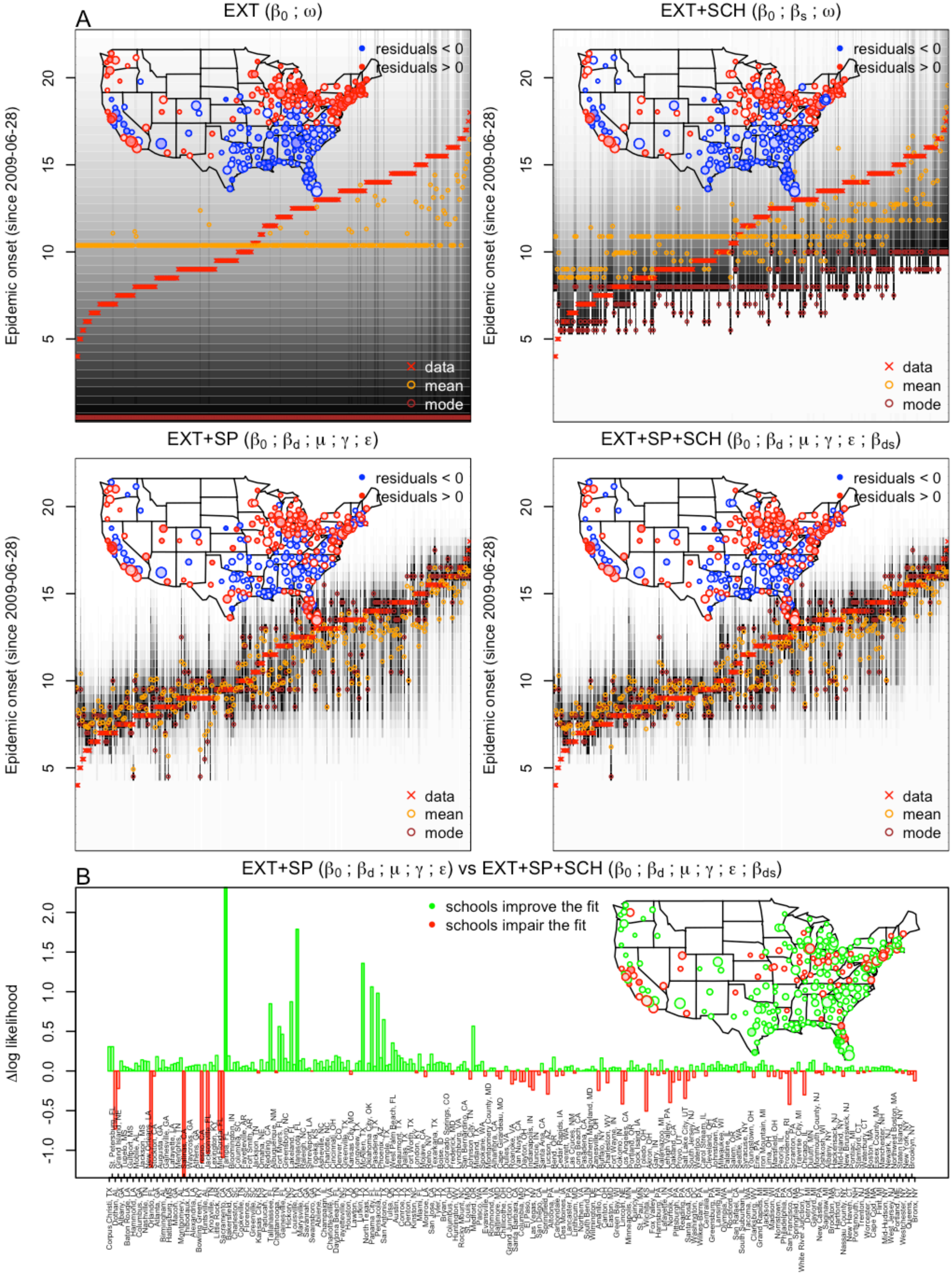

A
EXT (β_0 ; ω)
EXT+SCH (β_0 ; β_s ; ω)
EXT+SP (β_0 ; β_d ; μ ; γ ; ε)
EXT+SP+SCH (β_0 ; β_d ; μ ; γ ; ε ; β_{ds})
Epidemic onset (since 2009-06-28)
residuals < 0
residuals > 0
data
mean
mode
B
EXT+SP (β_0 ; β_d ; μ ; γ ; ε) vs EXT+SP+SCH (β_0 ; β_d ; μ ; γ ; ε ; β_{ds})
Δlog likelihood
schools improve the fit
schools impair the fit

Comparison of most parsimonious models to observed onset timings of the autumn 2009 pandemic

A: Conditional probability of epidemic onset, model prediction and residual analysis for the most parsimonious model per category. For each location (sorted by increasing time of onset) probabilities of epidemic onset conditional on the previous disease dynamic are represented in grey scale with mode in brown and model prediction (average) in orange. Residuals are computed as the difference between the model prediction (brown) and the data (red) and reported on the maps. Titles indicate parameters defining these models. Models discarding local spatial transmission are unable to reproduce the qualitative patterns of spread (upper panels). The inclusion of local spatial spread with or without school opening means the model broadly tracks the spatial progression of pandemic onsets (lower panels).

The best-fit model is able to reproduce the general spread pattern of the autumn 2009 pandemic wave originating from the South Eastern US, but the residuals are geographically clustered, particularly in California and Florida. Most notably, the model predicts later onsets than those observed in California's Central Valley and earlier onsets than observed in Florida.

B: While the spatial model with and without schools give broadly similar visual results, there is a significant improvement in model fit (see Table 2), and here this difference is investigated by location. For each location (x-axis and map) difference of log likelihood (conditional on the previous disease dynamic) between the model with and without schools are reported.
