# Supplementary material for: Spatial Transmission of 2009 Pandemic Influenza in the US
Source: PLoS Comput Biol. 2014 Jun 12;10(6):e1003635. doi: 10.1371/journal.pcbi.1003635 (PMC4055284; doi:10.1371/journal.pcbi.1003635)
Supplement: Figure S3 — Predictions from the most parsimonious model on the effect of school closure on pandemic onset timings of the autumn 2009 pandemic in the US. 1000 realisations were started without any locations infected. The full model (black lines) was simulated using maximum likelihood parameters of the most parsimonious model (see table 2). The simulations without school opening (grey lines) uses the same parameters, but schools were set as closed. The general spatial structure of the wave is similar to observed or simulated with the correct school opening times, but the spread was substantially slower. However, the exact length of the delay was sensitive to the fitted parameters. In the lower graphs, the spatial transmission parameter (βd) was fixed, other parameters refitted and the above simulations repeated. The 95% confidence intervals are indicated by black dashed lines and the maximum likelihood estimate by orange lines. The lower left plot shows the distribution of the time when 50% of the locations were infected (T50). The simulated times with schools (black boxes) was not sensitive to the fixed parameter, but the extra delay with schools closed (grey boxes) was highly variable over the confidence interval. The lower right graph shows how the transmission parameters are interdependent, which explains the sensitivity in simulation. In summary: the extent of the epidemic slowing from closing schools is difficult to estimate accurately from this model, but is likely to be substantial. (PDF) [file pcbi.1003635.s003.pdf]

Figure S3

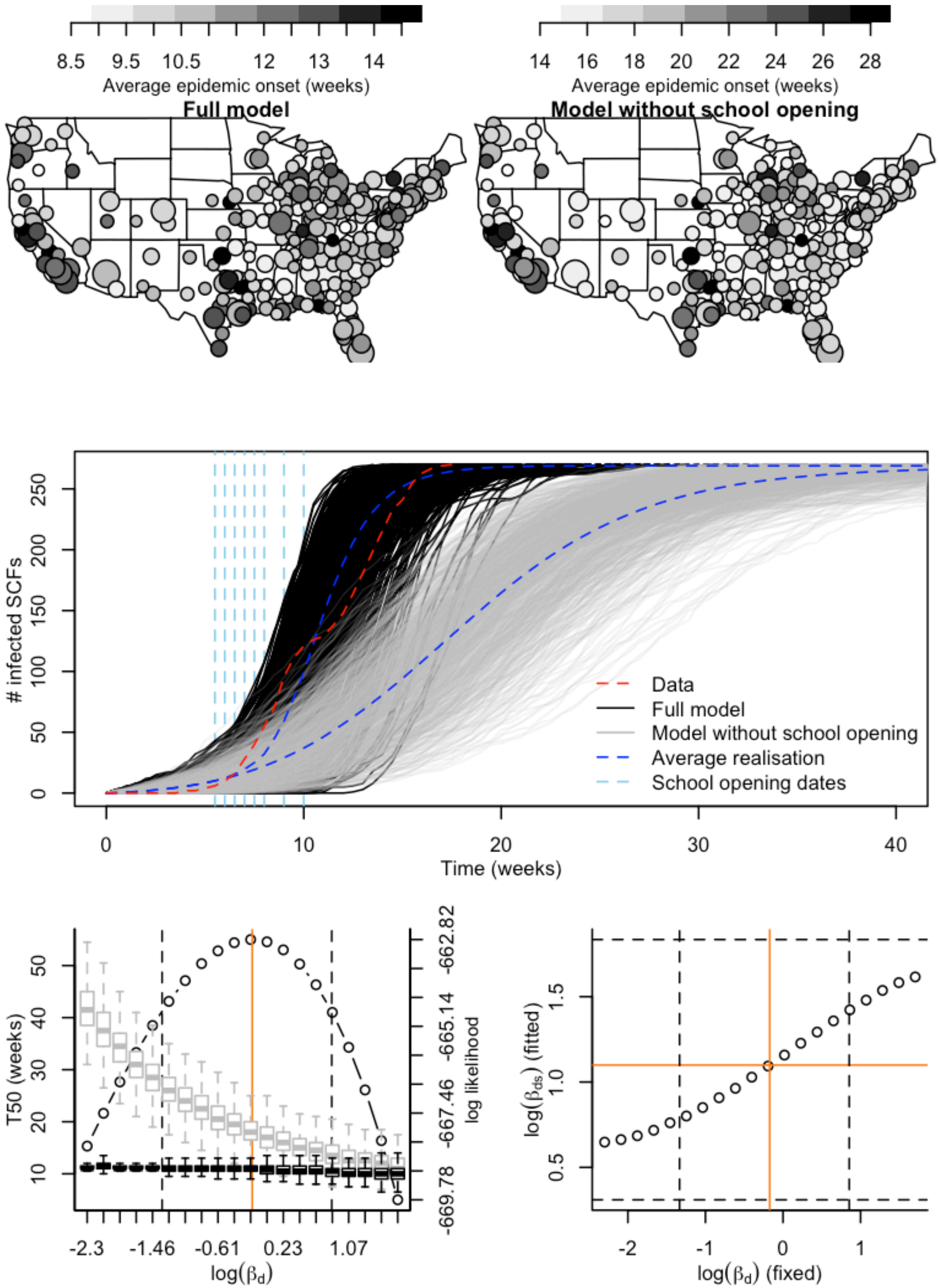
8.5 9.5 10.5 12 13 14
Average epidemic onset (weeks)
Full model
14 16 18 20 22 24 26 28
Average epidemic onset (weeks)
Model without school opening
infected SCFs
0 50 100 150 200 250
0 10 20 30 40
Time (weeks)
Data
Full model
Model without school opening
Average realisation
School opening dates
T50 (weeks)
10 20 30 40 50
-2.3 -1.46 -0.61 0.23 1.07
log(β_d)
-662.82 -665.14 -667.46 -669.78
log likelihood
log(β_ds) (fitted)
0.5 1.0 1.5
-2 -1 0 1
log(β_d) (fixed)

Predictions from the most parsimonious model on the effect of school closure on pandemic onset timings of the autumn 2009 pandemic in the US

1000 realisations were started without any locations infected. The full model (black lines) was simulated using maximum likelihood parameters of the most parsimonious model (see table 2). The simulations without school opening (grey lines) uses the same parameters, but schools were set as closed. The general spatial structure of the wave is similar to observed or simulated with the correct school opening times, but the spread was substantially slower. However, the exact length of the delay was sensitive to the fitted parameters. In the lower graphs, the spatial transmission parameter (β_d) was fixed, other parameters refitted and the above simulations repeated. The 95% confidence intervals are indicated by black dashed lines and the maximum likelihood estimate by orange lines. The lower left plot shows the distribution of the time when 50% of the locations were infected (T50). The simulated times with schools (black boxes) was not sensitive to the fixed parameter, but the extra delay with schools closed (grey boxes) was highly variable over the confidence interval. The lower right graph shows how the transmission parameters are interdependent, which explains the sensitivity in simulation. In summary: the extent of the epidemic slowing from closing schools is difficult to estimate accurately from this model, but is likely to be substantial.
