# Supplementary material for: Spatial Transmission of 2009 Pandemic Influenza in the US
Source: PLoS Comput Biol. 2014 Jun 12;10(6):e1003635. doi: 10.1371/journal.pcbi.1003635 (PMC4055284; doi:10.1371/journal.pcbi.1003635)
Supplement: Figure S4 — The profile likelihoods for the parameters in the most parsimonious model. For the transmission rate parameters (top row), logged parameter values are used, while the exponents (bottom row) are given unlogged. The orange line marks the maximum likelihood, and the dotted lines give the range for a drop of 1.92 in the log likelihood, corresponding to a 95% confidence interval. (PDF) [file pcbi.1003635.s004.pdf]

Figure S4

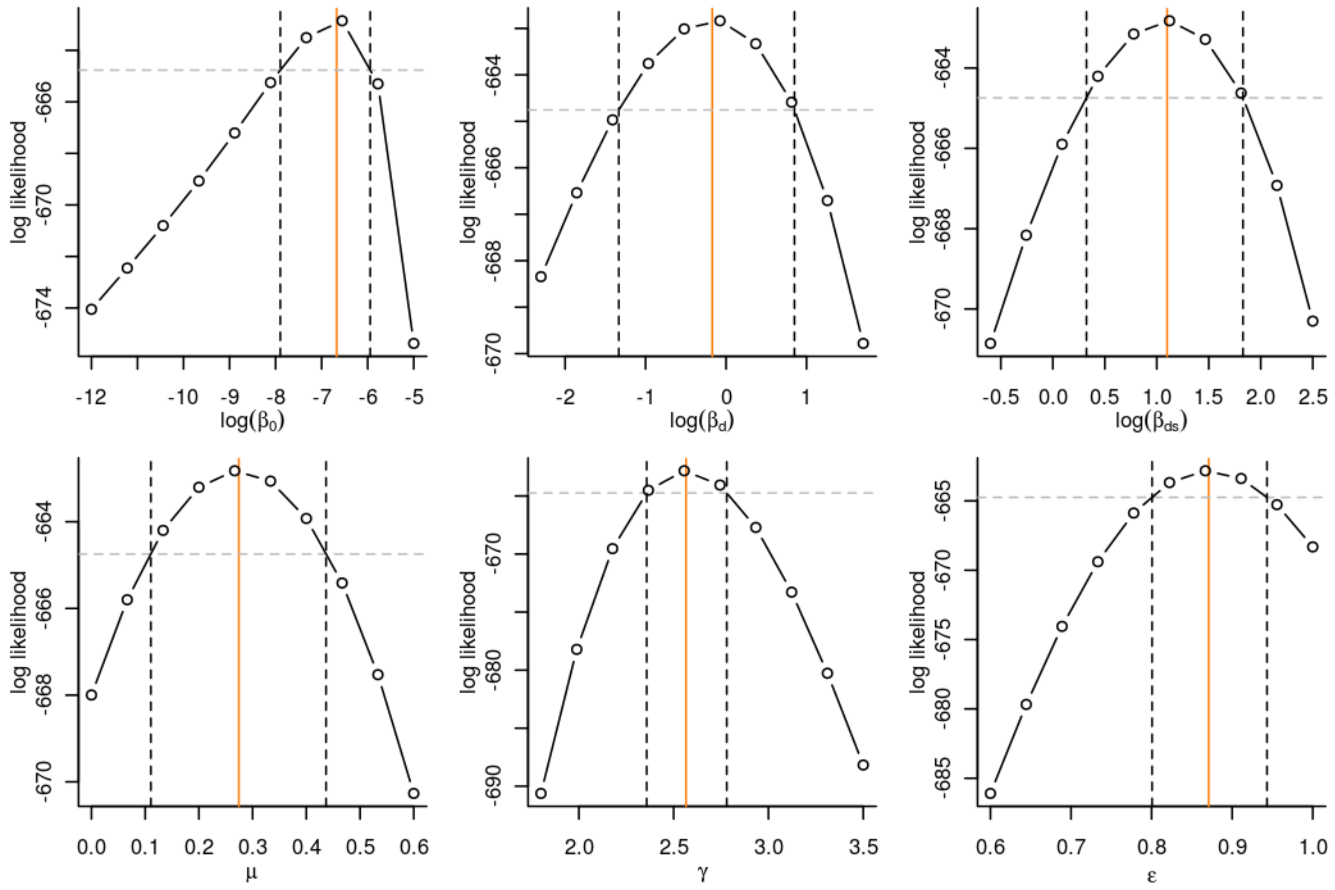

The profile likelihoods for the parameters in the most parsimonious model. For the transmission rate parameters (top row), logged parameter values are used, while the exponents (bottom row) are given unlogged. The orange line marks the maximum likelihood, and the dotted lines give the range for a drop of 1.92 in the log likelihood, corresponding to a 95% confidence interval.
