# Supplementary material for: Spatial Transmission of 2009 Pandemic Influenza in the US
Source: PLoS Comput Biol. 2014 Jun 12;10(6):e1003635. doi: 10.1371/journal.pcbi.1003635 (PMC4055284; doi:10.1371/journal.pcbi.1003635)
Supplement: Table S1 — Most parsimonious model per category including different spatial kernels. An extension of Table 2 from the main text: this table gives the log likelihood and AICc for the maximum likelihood fits to the most parsimonious models in each category. Here, additional results are given for the alternative spatial kernels (Gaussian, or using grid distance). For each model category, the most parsimonious model is specified by the parameters that are non-zero, which are given in the final column. In all cases, the gravity model has much lower AICc than the Gaussian or the grid models. Despite the crudeness of the spatial grid, the grid model performs surprisingly well. (PDF) [file pcbi.1003635.s005.pdf]

**Table S1: Most parsimonious model per category including different spatial kernels**

| Spatial model | Model Category | | | | Log Likelihood | AICc | ΔAICc | Parameters |
|---|---|---|---|---|---|---|---|---|
| - | EXT | | | | -1106.65 | 2217.35 | 879.39 | 2: $\beta_0, \omega$ |
| - | EXT | | | +AH | -1094.77 | 2195.63 | 857.67 | 3: $\beta_0, \omega, \varphi$ |
| - | EXT | | +SCH | | -860.77 | 1727.64 | 389.68 | 3: $\beta_0, \beta_s, \omega$ |
| - | EXT | | +SCH | +AH | -859.12 | 1726.40 | 388.44 | 4: $\beta_0, \beta_s, \omega, \varphi$ |
| Gravity | EXT | +SP | | | -675.06 | 1360.34 | 22.38 | 5: $\beta_0, \beta_d, \mu, \gamma, \varepsilon$ |
| Gravity | EXT | +SP | | +AH | -675.04 | 1362.04 | 24.08 | 6: $\beta_0, \beta_d, \mu, \gamma, \varepsilon, \varphi$ |
| **Gravity** | **EXT** | **+SP** | **+SCH** | | **-662.82** | **1337.96** | **0** | **6: $\beta_0, \beta_d, \mu, \gamma, \varepsilon, \beta_{ds}$** |
| Gravity | EXT | +SP | +SCH | +AH | -662.16 | 1338.75 | 0.79 | 7: $\beta_0, \beta_d, \mu, \gamma, \varepsilon, \beta_{ds}, \varphi$ |
| Gaussian | EXT | +SP | | | -688.10 | 1386.42 | 48.46 | 5: $\beta_0, \beta_d, \sigma_d, \varepsilon, \omega$ |
| Gaussian | EXT | +SP | | +AH | -687.90 | 1388.11 | 50.15 | 6: $\beta_0, \beta_d, \sigma_d, \varepsilon, \omega, \varphi$ |
| Gaussian | EXT | +SP | +SCH | | -681.10 | 1376.63 | 38.67 | 7: $\beta_0, \beta_d, \mu, \sigma_d, \varepsilon, \beta_{ds}, \omega$ |
| Gaussian | EXT | +SP | +SCH | +AH | -681.05 | 1378.65 | 40.69 | 8: $\beta_0, \beta_d, \mu, \sigma_d, \varepsilon, \beta_{ds}, \omega, \varphi$ |
| Grid | EXT | +SP | | | -694.20 | 1398.64 | 60.68 | 5: $\beta_0, \alpha, \beta_{d1}, \mu, \beta_{d2}$ |
| Grid | EXT | +SP | | +AH | -694.06 | 1400.45 | 62.49 | 6: $\beta_0, \alpha, \beta_{d1}, \mu, \beta_{d2}, \varphi$ |
| Grid | EXT | +SP | +SCH | | -678.13 | 1372.81 | 34.85 | 9: $\beta_0, \beta_s, \beta_{d1}, \mu, \beta_{d1s}, \beta_{d2}, \beta_{d2s}, \omega, \varphi$ |
| Grid | EXT | +SP | +SCH | +AH | -678.37 | 1373.29 | 35.33 | 8: $\beta_0, \beta_s, \beta_{d1}, \mu, \beta_{d1s}, \beta_{d2}, \beta_{d2s}, \varphi$ |

An extension of Table 2 from the main text: this table gives the log likelihood and AICc for the maximum likelihood fits to the most parsimonious models in each category. Here, additional results are given for the alternative spatial kernels (Gaussian, or using grid distance). For each model category, the most parsimonious model is specified by the parameters that are non-zero, which are given in the final column. In all cases, the gravity model has much lower AICc than the Gaussian or the grid models. Despite the crudeness of the spatial grid, the grid model performs surprisingly well.
