# Supplementary material for: Spatial Transmission of 2009 Pandemic Influenza in the US
Source: PLoS Comput Biol. 2014 Jun 12;10(6):e1003635. doi: 10.1371/journal.pcbi.1003635 (PMC4055284; doi:10.1371/journal.pcbi.1003635)
Supplement: Table S2 — Fitted parameters for the most parsimonious model. The most parsimonious model has six non-zero parameters. These are given in the table together with their maximum likelihood values and confidence intervals, as determined by a drop of 1.96 in the profile likelihoods. Setting the other parameters to zero, the force of infection for location i can be written as: . This force of infection is a rate and the units correspond to the time step Δt = half week. (PDF) [file pcbi.1003635.s006.pdf]

Table S2: Fitted parameters for the most parsimonious model

Parameter	Description	Units	Value	95% Confidence interval
β_0	Background transmission rate	$(\Delta t)^{-1}$	0.0013	0.0004-0.0028
β_d	Spatial transmission coefficient	$(\Delta t)^{-1}(km)^{1-\varepsilon}$	0.84	0.26-2.3
β_{ds}	Boost to β_d when schools are open	$(\Delta t)^{-1}(km)^{1-\varepsilon}$	3.0	1.4-6.3
μ	Exponent of dependence on recipient population size	none	0.27	0.11-0.44
γ	Exponent of distance in gravity model kernel	none	2.6	2.3-2.8
ε	Strength of density normalisation	none	0.87	0.80-0.94

The most parsimonious model has six non-zero parameters. These are given in the table together with their maximum likelihood values and confidence intervals, as determined by a drop of 1.96 in the profile likelihoods. Setting the other parameters to zero, the force of infection for location i can be written as:

$$\lambda_i(t) = \beta_0 + (\beta_d + \beta_{ds} I_i) N_i^{\mu} \frac{\sum_{j\in\Lambda} d_{i,j}^{-\gamma}}{\left[\sum_{j\neq i} d_{i,j}^{-\gamma}\right]^{\varepsilon}}$$

This force of infection is a rate and the units correspond to the time step Δt = half week.
